# Proof of Concept: Using ChatGPT to Teach Emergency Physicians How to Break Bad News

**DOI:** 10.7759/cureus.38755

**Published:** 2023-05-09

**Authors:** Jeremy J Webb

**Affiliations:** 1 Emergency Medicine, LewisGale Medical Center, Salem, USA; 2 School of Medicine, Edward Via College of Osteopathic Medicine, Blacksburg, USA

**Keywords:** medical education, breaking bad news, chatgpt, machine learning, chatbot, artificial intelligence

## Abstract

Background

Breaking bad news is an essential skill for practicing physicians, particularly in the field of emergency medicine (EM). Patient-physician communication teaching has previously relied on standardized patient scenarios and objective structured clinical examination formats. The novel use of artificial intelligence (AI) chatbot technology, such as Chat Generative Pre-trained Transformer (ChatGPT), may provide an alternative role in graduate medical education in this area. As a proof of concept, the author demonstrates how providing detailed prompts to the AI chatbot can facilitate the design of a realistic clinical scenario, enable active roleplay, and deliver effective feedback to physician trainees.

Methods

ChatGPT-3.5 language model was utilized to assist in the roleplay of breaking bad news. A detailed input prompt was designed to outline rules of play and grading assessment via a standardized scale. User inputs (physician role), chatbot outputs (patient role) and ChatGPT-generated feedback were recorded.

Results

ChatGPT set up a realistic training scenario on breaking bad news based on the initial prompt. Active roleplay as a patient in an emergency department setting was accomplished, and clear feedback was provided to the user through the application of the Setting up, Perception, Invitation, Knowledge, Emotions with Empathy, and Strategy or Summary (SPIKES) framework for breaking bad news.

Conclusion

The novel use of AI chatbot technology to assist educators is abundant with potential. ChatGPT was able to design an appropriate scenario, provide a means for simulated patient-physician roleplay, and deliver real-time feedback to the physician user. Future studies are required to expand use to a targeted group of EM physician trainees and provide best practice guidelines for AI use in graduate medical education.

## Introduction

Chat Generative Pre-trained Transformer (ChatGPT) is a free-use artificial intelligence (AI) chatbot. Developed by OpenAI, it was trained on 570 gigabytes of data and over 300 billion words. It has the ability to generate human-like responses to user inputs and uses Reinforcement Learning with Human Feedback (RLHF) to optimize its responses [1]. Machine learning algorithms make it possible for the chatbot to interact with individual users and provide informed responses in a conversational style. It also learns from these interactions and can adapt its future responses to past exchanges.

Since it first launched in November 2022, users have been experimenting with the chatbot to perform tasks other than simple chat. Some examples include using ChatGPT to write college essays, play games, provide advice and tips on topics of interest, solve complex math problems, and write computer code [2,3].

Novel uses of ChatGPT have been documented in the medical literature. These include outlining medical journal submissions, scripting patient education, and answering questions regarding complex medical topics [4,5]. It has even passed the Step 1 United States Medical Licensing Exam [6]. Some authors have theorized that the technology may be used in graduate medical education, especially as a roleplaying device to generate human-like responses in fictional but realistic clinical scenarios [7].

There is literature to support the practice of utilizing AI chatbots to train healthcare workers’ conversation skills [8]. Another recent study discovered that ChatGPT could deliver higher quality and more empathetic responses compared to physicians and outlined its potential use as a tool to help physicians deliver important communication to patients [9]. There are also published reports in the emergency department (ED) setting of using AI as a tool to improve patient-physician interaction and aid triage assessment [10,11]. However, there are no published reports of utilizing ChatGPT as an educational tool to train ED physicians in the difficult and emotional conversation of breaking bad news to patients.

Breaking bad news is one of the more challenging clinical encounters in medicine, especially in the ED, where patients may receive sudden and devastating news about their health. The way this news is delivered can impact patients’ well-being, mood, and satisfaction with their medical care [12]. While breaking bad news is an essential skill for physicians, it can also be emotionally taxing. Many doctors feel unprepared to handle this task, even though they recognize its significance [13]. Fortunately, studies have shown that formal training can lead to improved self-perception and performance in this area [14].

One effective educational approach includes a combination of formal didactics, roleplay, and simulated patient encounters. This method has been considered to be an ideal way to teach the skill of breaking bad news [15]. The author’s aim in this study was to see if ChatGPT could perform these tasks, by building a scenario in which an emergency physician could roleplay with a simulated patient to break bad news and provide didactic feedback to the user in real time.

## Materials and methods

**Figure 1:**
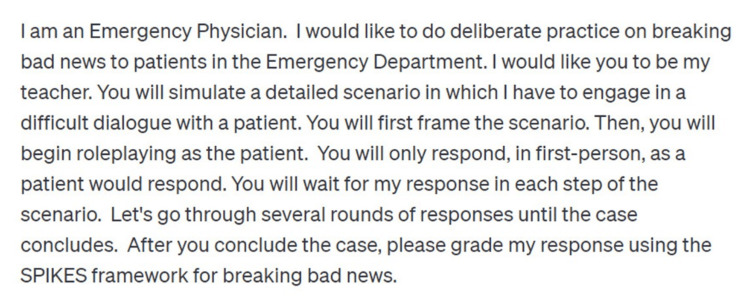
Initial user input prompt.

The ChatGPT-3.5 language model was accessed through the free-use OpenAI website (https://chat.openai.com/chat). An initial input prompt was modeled after a simulated business negotiation created by Ethan Mollick, an associate professor at Wharton University [16]. The input prompt (Figure 1) was edited to include information about the type of scenario the author wished to simulate (breaking bad news) and the role the author wanted ChatGPT to play (the patient). Stepwise tasks were assigned to the chatbot to ensure roleplay and grading via a standardized and highly studied framework for breaking bad news, known as Setting up, Perception, Invitation, Knowledge, Emotions with Empathy, and Strategy or Summary (SPIKES) [17]. Playing the role of the physician, additional inputs were typed by the author in response to ChatGPT’s output to simulate a natural conversation in an ED setting. Input and output responses were recorded via the software screenshot tool.

## Results

**Figure 2:**
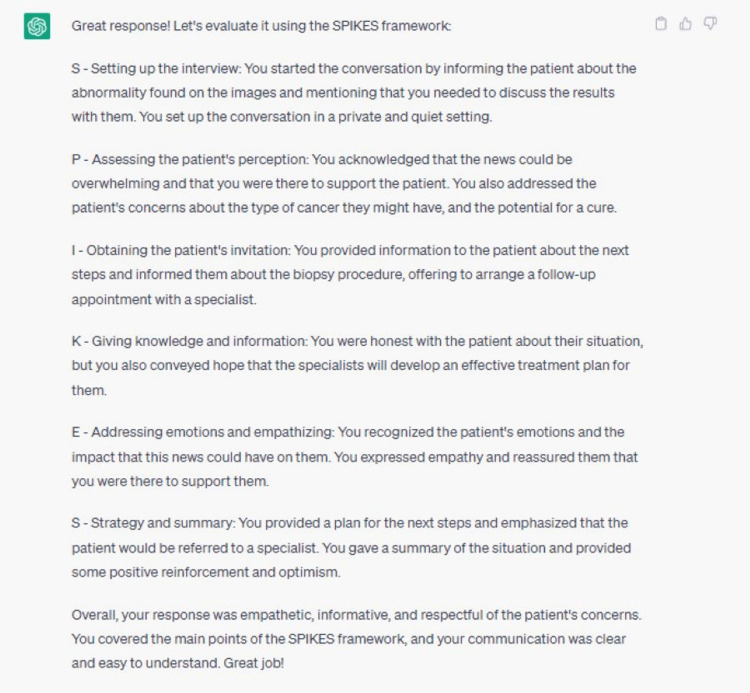
ChatGPT final output. ChatGPT: Chat Generative Pre-trained Transformer

Input and output data are recorded in Table 1. ChatGPT set up a scenario based on the initial prompt and initiated a simulated conversation by roleplaying as a patient. The chatbot allowed time for an input response and responded in character. ChatGPT prompted user input by asking pointed questions throughout the encounter. At the scenario’s completion, the chatbot utilized the standardized SPIKES framework for breaking bad news to provide detailed feedback (Figure 2).

**Table 1:** User input and ChatGPT output responses during roleplay. ChatGPT: Chat Generative Pre-trained Transformer

User input	ChatGPT output
Initial input prompt (Figure 1)	Sure, I can help you with that. Here’s a scenario we can use. Scenario: You are an emergency physician, and I am a patient who has been brought to the emergency department with severe abdominal pain. You’ve just finished the initial assessment and tests and have come to the conclusion that the patient has pancreatic cancer. Patient: Hello, doctor. What did the tests show? Is everything okay? (Your turn to respond.)
I wanted to talk with you about some of the testing that has come back. As you know, we obtained some images of your belly to know exactly what is causing your pain. After reviewing the pictures, we have discovered an abnormality. The radiologist has identified a growth near your pancreas that is very concerning for a cancer.	Patient: Cancer? Are you serious? How can this be happening to me? I feel fine. How long do I have to live? Can you cure me? (Your turn to respond.)
I understand this can be overwhelming to hear. This news can be frightening. I want to let you know we are here to support you through this. It is too early to know exactly what kind of cancer you have at this time. You will need further testing to help us identify the exact type of cancer this is. Once we have figured this out, the specialists can decide the best course of treatment. The next step in diagnosis will be a biopsy procedure, where a specialist will try to obtain a small piece of the growth in order to run further tests on it. We can arrange follow-up with the specialist this week to get this done.	Patient: Okay, I understand. But what are my chances of survival? How long do I have to live? Can this cancer be cured? (Your turn to respond.)
It really depends on the exact type of cancer you have. Some cancers do really well with treatments such as chemotherapy, radiation, and surgery. There are many people who present similarly to your case, who do very well. Some cancers are harder to treat. It is difficult to know without further testing, but I’m optimistic our specialists will come up with a good treatment plan for you.	Final output (Figure 2)

## Discussion

The overall performance of ChatGPT to both roleplay with and provide education on breaking bad news was good. In the example above, it appeared that the training dataset utilized by ChatGPT was sufficient to enable task performance assigned in the initial user prompt. The feedback was constructive and highlighted aspects of the user’s approach. As prompted, the user’s responses were assessed using the standardized SPIKES framework for breaking bad news. The chatbot was also able to successfully recall specific details of the roleplay for each area evaluated.

Although in its infancy, AI chatbot use has the potential to disrupt how we teach medical students and graduate medical residents communication skills in outpatient and hospital settings [18]. Chatbots may provide wider access to patient-physician roleplaying scenarios by substituting for human standardized patients, which carry a cost and personnel burden. Users may rely on scenarios ChatGPT builds itself, as in the case above, or input a specific clinical scenario they want to practice. Also, AI-guided instruction may be accomplished in locations outside the traditional hospital or training location due to the ubiquity of internet access.

There are several limitations to this study, and more rigorous testing methods and analyses will be needed to fully assess the strengths and weaknesses of AI use for roleplay training in medical education. This is an observational study with a single ideal conversation recorded. Therefore, feedback was generally positive. It may be interesting to input suboptimal physician roleplay inputs to see if appropriate negative feedback was provided. It would also be interesting to see if general feedback differs with a user level of training (e.g., medical student, intern, senior resident, early career attending, and later career attending).

Technically, an individual AI chatbot’s performance is limited by the dataset on which it learns as well as the prompts it receives from users. For example, even though it was trained on a large dataset, ChatGPT has limited knowledge past September 2021 because training data beyond that time was not included [19]. New research beyond that date regarding advances in communication theory, empathic conversational tools, and medical knowledge is limited. Prompt design is also very important. Refinement of user input to elicit quality and detailed chatbot output is essential, especially when testing emergent capabilities. Some authors have termed such user input a SuperPrompt [20]. Additionally, there are instances where ChatGPT provides inaccurate responses to user inputs [21], and it cannot assess or relay the intangibles of human communication, such as eye contact, pausing to listen, and tone.

In the future, it may be possible to build a more robust educational chatbot trained on a dataset specifically dealing with medical pathology, treatment options, clinical outcomes, psychology of conversation, and patient satisfaction. A higher fidelity experience may also be created by combining an AI chatbot with additional technology. Text-to-speech function, speech pattern recognition, avatar use, and virtual reality might enhance the efficacy of educational roleplay [22,23].

A more detailed and extensive follow-up study is essential to evaluate ChatGPT’s proficiency in roleplaying physician delivery of bad news. Several methods could be used to achieve this goal. For instance, the chatbot could be utilized in a manner similar to the techniques described above, with prompt assignment by a physician faculty member. Emergency medicine residents would then engage with the chatbot and receive feedback on their written responses. Alternatively, a hybrid objective structured clinical examination (OSCE) format could be employed that combines AI technology with in-person roleplay. In this case, a faculty member would read ChatGPT’s outputs as a script in conversational style while trainees respond with natural speech. An assistant can transcribe the roleplay into the chatbot in real time, which may lead to a more natural interaction. The use of pre- and post-surveys would help gather feedback from faculty and trainees on the chatbot’s effectiveness as a training tool, its ease of use, and the quality of feedback it provides. In addition, the use of AI could facilitate repetitive self- or peer-led training throughout the academic year. Such an approach could be cost-effective and without significant administrative burden and enhance exposure throughout the training period [24].

## Conclusions

The use of AI chatbots to provide education to medical trainees and postgraduate physicians appears abundant with potential. ChatGPT has illustrated how it can create a realistic training scenario, roleplay in a simulated patient-physician text-based exchange, and grade individual user performance. Although more robust research is needed to flesh out best-use practices and identify technological shortcomings, this author expects to see a rise in AI use and machine learning for medical education purposes and patient-physician communication training.
